# Impact of Prenatal Health Conditions and Health Behaviors in Pregnant Women on Infant Birth Defects in the United States Using CDC-PRAMS 2018 Survey

**DOI:** 10.3390/pediatric15010015

**Published:** 2023-03-01

**Authors:** Girish Suresh Shelke, Rochisha Marwaha, Pankil Shah, Suman N. Challa

**Affiliations:** 1 Helping Restore Ability, Arlington, TX 76018, USA; 2 School of Dentistry, University of Texas Health Science Center San Antonio, University of Texas, San Antonio, TX 78229, USA; 3 Department of Urology, University of Texas Health Science Center San Antonio, University of Texas, San Antonio, TX 78229, USA

**Keywords:** pregnancy, birth defect, national, PRAMS (Pregnancy Risk Assessment Monitoring System), smoking, diabetes, depression

## Abstract

Objective: To assess both individual and interactive effects of prenatal medical conditions depression and diabetes, and health behaviors including smoking during pregnancy on infant birth defects. Methods: The data for this research study were collected by the Pregnancy Risk Assessment Monitoring System (PRAMS) in 2018. Birth certificate records were used in each participating jurisdiction to select a sample representative of all women who delivered a live-born infant. Complex sampling weights were used to analyze the data with a weighted sample size of 4,536,867. Descriptive statistics were performed to explore frequencies of the independent and dependent variables. Bivariate and multivariable analyses were conducted to examine associations among the independent and dependent variables. Results: The results indicate significant interaction between the variables smoking and depression and depression and diabetes (OR = 3.17; *p*-value < 0.001 and OR = 3.13; *p*-value < 0.001, respectively). Depression during pregnancy was found to be strongly associated with delivering an infant with a birth defect (OR = 1.31, *p*-value < 0.001). Conclusion: Depression during pregnancy and its interaction with smoking and diabetes are vital in determining birth defects in infants. The results indicate that birth defects in the United States can be reduced by lowering depression in pregnant women.

## 1. Introduction

Birth defects are a primary cause of fetal death, infant mortality and morbidity, and long-term disability. Birth defects affect the quality of life of these infants and pose a burden for their families and society. According to the World Health Organization (WHO), about 300,000 newborns diagnosed with birth defects die within the first 28 days of life [1]. Approximately 3.3% of live births in the United States constitute severe birth defects [1]. Birth defects result in an increased cost of care for children born with a birth defect compared to those with no birth defects [2]. The increased cost of care affects access to oral health care for children with birth defects [2]. Birth defects represent a significant public health issue due to their long-term individual and social consequences.

Diabetes mellitus (DM) is a metabolic disease that results in hyperglycemia and is caused by either the low level of insulin in the body or resistance to insulin [3]. The overall prevalence ratio of offspring with any form of birth defects in women with pre-existing diabetes is 5.88% compared to women without diabetes or gestational diabetes [4]. In pregnant mothers, Type 1 and Type 2 diabetes show a stronger association with craniofacial abnormalities in offspring, indicating a prevalence ratio of 8.9 compared to non-diabetic women [4].

Several studies indicate that birth defects are associated with maternal smoking [4,5]. In a systematic review of 38 studies, 13 studies indicated a significant association between smoking and orofacial clefts with a pooled odds ratio of 1.28, and 6 studies revealed a dose-response relationship [6,7]. Another meta-analysis using 29 case-control and cohort studies could not detect a dose-response relationship; however, it indicated a moderate risk of birth defects associated with smoking during pregnancy with an odds ratio of 1.29 [7].

Likewise, prenatal depression among pregnant women poses a comprehensive public health problem and is a potential risk factor for adverse birth outcomes [8]. A cross-sectional study conducted in Wuhan, China, between March 2013 and April 2014 suggested that prenatal depression was significantly associated with birth defects. The adjusted odds ratio for this variable was 1.67 compared with women reporting no prenatal depression; however, no temporal relationship could be established since it was a cross-sectional study [8,9]. This reflects an association between maternal depression and birth defects [8]. The study concluded that reducing the maternal depression can significantly reduce the risk of birth defects [8].

Although previous studies have addressed the impact of prenatal health conditions and health behaviors on birth defects, they did not assess the interactive effect of these variables on birth defects. This study aims to address the gaps in the literature by understanding both the individual and interactive effects of smoking during pregnancy, diabetes, and prenatal depression on birth defects. The study aims at finding the risk of delivering a child with birth defect in the women with depression, diabetes, and health behaviors such as smoking.

The primary objective of this research was to assess both the individual and interactive effects of prenatal depression, diabetes, and smoking in pregnant women on infant birth defects. The study hypothesized that birth defects are associated with two-way or three-way interactions of prenatal depression, smoking, and diabetes during pregnancy.

## 2. Materials and Methods

The research proposal was approved by Institutional Review Board at U.T. Health San Antonio on 2 March 2021. The IRB number is HSC20210029N. This secondary research planned to analyze the data collected through the Pregnancy Risk Assessment Monitoring System (PRAMS) survey datasets. PRAMS is a joint research project between the state, territorial, or local health departments and the Centers for Disease Control and Prevention, Division of Reproductive Health. The PRAMS survey dataset is a multistate analytic dataset created by the stratified sampling technique [10]. A sample of women across all PRAMS sites in the United States who had a recent live birth was collected from the state’s birth certificate file for the PRAMS survey [11].

The dataset contains demographic and clinical information collected through the state’s vital records system, birth certificate, and other variables such as operational, weighting, questionnaire, and analytic variables. Topics addressed in the PRAMS survey questionnaire included prenatal care, obstetric history, maternal habits, physical abuse, contraception, economic status, maternal stress, and early infant development. Each year, the data are collected through surveys and are available publicly after 14 months for a specific year. This study used the PRAMS data collected for the year 2018. The birth defect information is retrieved by the PRAMS through the birth certificate record and linked with the survey responder. The data received from PRAMS include information about birth defects which is classified as the binary variable of Yes or No. This birth defect binary variable includes all the birth defect-related anomalies [11].

The Pregnancy Risk Assessment Monitoring System (PRAMS) combined two modes of data collection, which were a survey conducted by mailed questionnaire with multiple follow-ups and a telephone survey [8]. Overall, 89,839 US women who had a recent live birth responded to the PRAMS mail questionnaire or participated in the PRAMS phone survey [12]. These 89,839 women were included as respondents in the present research [11]. Non-respondents to the questionnaires were excluded from the study reported in this paper. California, Idaho, and Ohio did not participate in the CDC-PRAMS 2018 survey. Hence, the data for these states were not available.

The data obtained from CDC-PRAMS for 2018 were used to create a new data subset for analysis following initial data cleaning and merging. The independent or exposure variables included prenatal conditions and health behaviors such as depression, smoking, and diabetes. The birth defect variable, dependent or outcome variable, was dichotomous and classified as “Yes” for the presence of a birth defect and “No” for the absence of a birth defect. The “diabetes during pregnancy variable” classification is different. The PRAMS dataset combined all the types of diabetes together including Type 1, Type 2, and gestational diabetes. The diabetes during pregnancy variable in this dataset is binary showing “Yes” and “No”. The pregnant mother who responded yes reflects diabetes which includes Type 1, Type 2, and gestational diabetes. The data were analyzed using SPSS, Version 26 (SPSS, 2020).

Univariate analyses were used to explore the frequencies for the dependent, independent, and demographic variables. Chi-square tests were conducted to test the associations between the birth defect variable with independent variables such as smoking, depression, and diabetes. Subsequently, the logistic regression model was used for diabetes during pregnancy and testing the interactive effects among two or more covariates. The effect modification was determined in the logistic regression model. A multiplicative model was the model of choice to determine the interactive effect of smoking, depression, and diabetes variables, and to assess various risk factors of birth defects, including depression, smoking during pregnancy.

## 3. Results

The average survey response rate of the PRAMS survey for all states was 56.81%. The total sample of respondents is 89,839. This sample size is all the respondents of the study survey. After applying the complex sampling weight to the survey data, the total sample size is 4,536,867.

**Figure 1:**
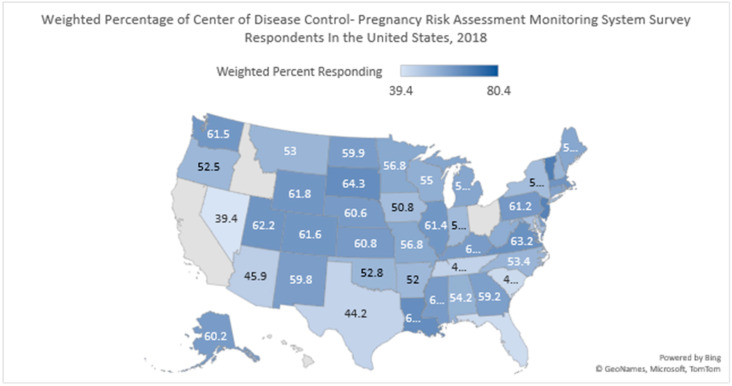
The weighted survey response rate according to different states. Note: The figure represents the weighted survey response rate for all the states in the United States. Each number corresponds to the percentage weighted survey response rate.

The response rate varied from as high as 80.4% for Puerto Rico to as low as 39.4% for Nevada (Figure 1). After applying complex sampling, out of 4,536,867 live births in 2018, birth defects were reflected in 0.3% of the total live births (Table 1).

The initial data suggest that the study population varied by age, race, body mass index, and other health indicators. Women who were 25 to 34 years of age contributed to about 58.30% of infants born with birth defects, with most pregnant women being White (67%) and having an income of USD 57,000 to 85,000 (38.6%). The data indicate that 7.2% of pregnant women smoked during pregnancy, 14.2% were diagnosed with depression, and 9.6% were diagnosed with diabetes. The bivariate analysis revealed statistically significant Chi-square values for demographic variables, including age (*p*-value < 0.001), race (*p*-value < 0.001), body mass index (*p*-value < 0.001), maternal smoking habits during pregnancy (*p*-value < 0.001), depression during pregnancy (*p*-value < 0.006), diabetes during pregnancy (*p*-value < 0.023), abuse (*p*-value < 0.001), folic acid intake (*p*-value < 0.001), vitamin intake (*p*-value < 0.001), and hypertension (*p*-value < 0.001) (Table 1).

The multivariate binary logistic regression model for the PRAMS data indicates that age, race, depression during pregnancy, maternal smoking, abuse during pregnancy, hypertension, and smoking e-cigarettes are significantly associated with birth defects (Table 2). The study result indicated that the birth defect did not vary much according to the income category when other variables are constant, and the change is the odds ratio is small to determine any association. (Table 2).

After adjusting the model for covariates, Asian women were at a higher risk of birth defects compared to White women (*p*-value <0.001, OR = 1.17). The women in the age group of 25–29 years show lower odds of delivering a child with birth defect compared to maternal age groups of 18–19, 20–24, and 40 years and above (OR = 1.67, *p*-value < 0.001; OR = 1.24, *p*-value < 0.001; and OR = 2.20; *p*-value < 0.001, respectively).

The odds of delivering an infant with a birth defect in mothers who smoke during pregnancy are approximately two times the odds for mothers who do not smoke during pregnancy (OR = 2.29, *p*-value < 0.001). Depression during pregnancy was significantly associated with infants born with birth defects with odds of 1.31 compared to women with no depression during the prenatal period (OR = 1.31, *p*-value < 0.001). Diabetes during pregnancy was negatively associated with birth defects compared to non-diabetic women when the model was controlled for covariates. (OR = 0.68; *p*-value < 0.001 and OR = 0.75; *p*-value < 0.001, respectively).

**Figure 2:**
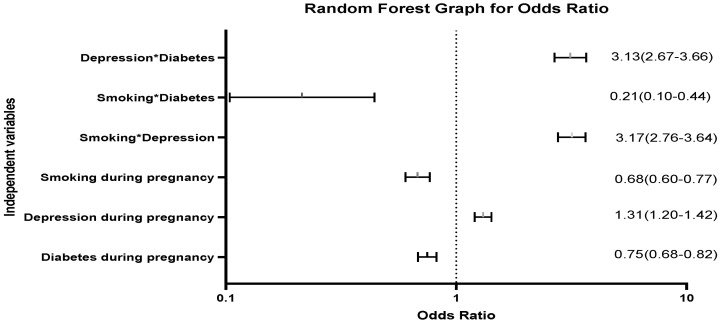
Random forest plot for odds ratio. Note: The figure represents the random forest graph for the odds ratio and corresponding confidence intervals. * Represents the interaction between two variables. 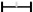 Represent the confidence interval of odds ratio.

**Figure 3:**
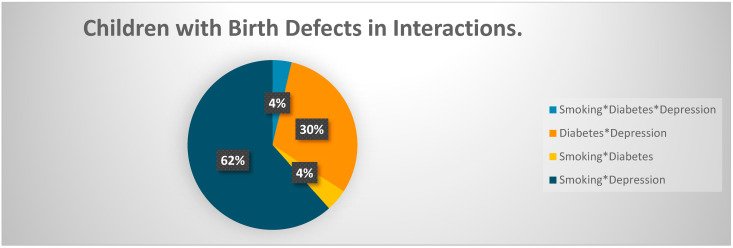
Children with birth defects in interactions. Note: The figure represents children with birth defects when only the interaction of smoking, diabetes, and depression is plotted. * Represent interaction between variables.

The interaction of smoking, diabetes, and depression during pregnancy was insignificant in determining birth defects in children (*p*-value = 0.966). The interaction of smoking and depression resulted in higher odds of delivering the child with a birth defect compared to mothers who did not report smoking or depression when a multivariable model was controlled for covariates (OR = 3.17; *p*-value < 0.001) (Figure 2: Random Forests plot for the odds ratio and Figure 3). The interaction of depression and diabetes in pregnant women resulted in odds of 3.13 for delivering a baby with a birth defect compared to women with no depression and diabetes in the controlled multivariable model (OR = 3.13; *p*-value < 0.001). The interaction of smoking and diabetes was negatively associated with birth defects when other variables were constant (OR = 0.21; *p*-value < 0.001) (Figure 2 and Figure 3).

## 4. Discussion

This research to analyze both individual and interactive effects of smoking, diabetes, and depression during pregnancy on birth defects using recent data from all PRAMS active sites in the United States for 2018 [11]. The complex sampling methods used for analysis provided a representative sample for all pregnant women in the United States in 2018 [11].

The interaction of all three variables, smoking, diabetes, and depression, during pregnancy, was insignificant (*p*-value = 0.966) in predicting birth defects in children. The findings are inconsistent with the study hypothesis that these variables show an interactive effect in determining birth defects, thus rejecting this hypothesis. Insignificant results could be due to different reasons, such as study design and selection bias. The sample of women who answered yes to smoking, diabetes, and depression during pregnancy was very small to identify the effect of exposure in the population (Table 1 and Table 2). There may be response bias for this question as pregnant women most likely misreported smoking behavior and depression during pregnancy.

The strength of this study was its large sample size and reasonable survey response rate. This study was based on recent data collected for CDC-PRAMS, which provided the latest results for variables of interest. The average survey response rate for CDC-PRAMS was 56.81% [1] (Figure 1). The large sample size and adequate response rate supports the external validity of this study. The analysis weights used in the study were calculated from sampling, non-response, and non-coverage weights, which represented other women similar to respondents in the sample [1]. The study results need to be tested with other equivalent populations such as Europe or Asia to test the study’s external validity further. The sample used for this study effectively analyzed the strata with fewer participants, reflecting good internal validity. The stratified systematic sampling technique and weighting reduced sampling and selection bias, respectively which improved the study’s internal validity. Although the study design was cross-sectional, minimizing the selection bias and sampling techniques enhanced the study’s internal validity.

This study provided more evidence supporting the interaction of depression and diabetes as well as smoking and depression on birth defects compared to the other variables. The interaction presented in the study might be due to the confounding effect of the depression variable, and this variable should be adjusted to analyze the interaction of smoking and diabetes with depression during pregnancy. Previous studies have depicted the effect of the single variable of interest, i.e., smoking, diabetes, and depression during pregnancy alone, on the outcome variable [4,13,14,15,16]. This study of the interaction was unique as it analyzed nationwide data to understand the interactive effect of smoking, diabetes, and depression during pregnancy on a rare event such as a birth defect.

The results of this study indicated that depression in pregnant women is related to birth defects in infants, which is consistent with previous studies suggesting that depression during pregnancy may be due to domestic violence and abuse. Yu and colleagues reported a significant impact of domestic violence, i.e., abuse (OR = 1.67) and depression (OR = 1.72) in pregnant mothers, on delivering children with birth defects [8]. The result of our study confirms the association of maternal depression with the birth defect and is consistent with the prior studies.

Previous studies did not analyze the interaction of smoking and depression, but the present study provided significant evidence of the interactive effects of smoking and depression during pregnancy on children with birth defects (OR = 3.17; *p*-value < 0.001). This study used a cross-sectional study design and larger sample, which provided the necessary number of subjects to analyze the effect of smoking and depression on birth defects. Another prospective cohort study proposed that diabetes, including gestational diabetes and its interaction with obesity, was significantly related to birth defects and increased birth defects by 65% [9]. Our study used classification criteria for diabetes similar to those used by Moore et al. (2000b), but reached contradicting results [3]. These contradicting results might be due to lower cases of pregnant women diagnosed with Type 1 and Type 2 diabetes. Gestational diabetes cases might have contributed more to the sample of diabetes, which resulted in contradicting results as gestational diabetes does not have a stronger association with birth defects [9]. This study used the already collected survey data by CDC-PRAMS, so it was not possible to separate the diabetes cases in gestational or non-gestational diabetes. For future studies, this variable should be reclassified to understand the impact of different types of diabetes on birth defects.

The study shows that income distribution is not a strong factor in determining the birth defect. The income status predicts health care utilization in rural areas. The recent research completed by Shelke et al. suggests that preventive treatment utilization is improved in rural areas and more in the income category of USD 25,000 to 45,000 compared to other income groups [17]. The income-related differences did not show a strong odds ratio in the current study which depicts that healthcare access is improving and pregnant women in all age groups are getting access to healthcare.

This study proposed a new direction of analyzing different variables and interactions to evaluate the effect on the birth defects. The study confirmed the impact of depression in pregnant women on birth defects. Further research can assess the effects of depression and conditions leading to depression during pregnancy on delivering a child with birth defects. Positive mental health and reducing abuse that leads to depression can help reduce birth defects in most US populations.

A limitation of this study is the use of the cross-sectional study design, which restricted the establishment of temporality between the independent variables of smoking, diabetes, and depression during pregnancy on the birth defect. The data were self-reported, which might lead to information bias, which was reflected in a smaller sample size of subjects reporting health behaviors such as smoking. Another limitation included no participation from a few states in the CDC-PRAMS 2018 survey (Figure 1). The income variable was classified using more than one classification system that imposed overlapping categories and challenges in reclassifying this variable in rational categories. The few subcategories of race variables such as Chinese, Japanese, Filipino, Hawaiian, American Indian, and Alaskan Native showed minimal participation, resulting in a higher standard error in regression analysis. This variable was then reclassified into “Other Asian” and “Other American Including Tribes”. The diabetes variable should have been classified as Type 1, Type 2, and gestational diabetes to understand the impact on birth defects. This is one of the limitations of this paper, and we did not succeed in determining the impact of the different types of diabetes on the birth defect.

The CDC-PRAMS data did not classify birth defects into different categories. Individual PRAMS sites collected the birth defect variables, but data collection from birth records varies from state to state. All PRAMS data collection sites were contacted to retrieve this information to gather data on types of the birth defect but did not receive information on different types of birth defects. States restrict the sharing of birth defect information because it is an extremely rare anomaly with very few reported cases annually and may violate PHI (Protected Health information) or HIPAA (Health Insurance Portability and Accountability Act) possibility due to linkage of birth defect data individually with birth records. If given a chance to repeat this study, more emphasis should be placed on understanding the individual and interactive impact of different types of birth defects.

## 5. Conclusions

This study provided strong evidence that depression during pregnancy is associated with birth defects and variables leading to depression, including abuse or other mental health issues that can be related to childbirth defects [3,9]. The current study highlights the need to understand the causal relationship between depression during pregnancy and birth defects.

The awareness of depression and its possible impact on birth defects in children should be prioritized. More efforts are needed to educate pregnant women about managing stress, reporting abuse, and maintaining mental health to avoid depression during pregnancy. To mitigate birth defects, federal programs for maternal and child health, including HRSA (Health Resources and Services Administration) grants “Maternal, Infant, and Early Childhood Home Visiting Program”, should focus more on providing resources for depression during pregnancy and conditions responsible for depression [18,19].

## Figures and Tables

**Table 1:** Sociodemographic information of pregnant women; the United States, 2018.

Variables	Data Characteristics		Birth Defect			
	N ^‡^	Percentage	Yes N ^‡^ (%)	No N ^‡^ (%)	Chi-Square ^§^	*p*-Value ^†^
Mother’s characteristics						
Age						
<17	50,364	1.1%	98 (0.2)	50,229 (99.8)	800.13	<0.001
18–19	139,179	3.1%	399 (0.3)	138,374 (99.7)		
20–24	845,587	18.6%	3093 (0.4)	841,067 (99.6)		
25–29	1,314,183	29.0%	2956 (0.2)	1,308,909 (99.8)		
30–34	1,328,787	29.3%	3230 (0.2)	1,320,862 (99.8)		
35–39	703,497	15.5%	1653 (0.2)	700,054 (99.8)		
40+	155,236	3.4%	791 (0.5)	153,818 (99.5)		
Missing	34	0.0%				
Income						
Zero–USD 28,000	1,508,760	33.3%	4255 (0.3)	1,500,510 (99.7)	34.9	<0.001
USD 28,000–57,000	879,645	19.4%	2269 (0.3)	875,240 (99.7)		
USD 57,000–85,000 or More	1,751,664	38.6%	4362 (0.2)	1,743,236 (99.8)		
Missing	396,795	8.7%				
Race						
Asian	229,932	5.1%	666 (0.3)	228,766 (99.7)	198.11	<0.001
Other American Including Tribes	38,887	0.9%	75 (0.2)	38,749 (99.8)		
Black	757,121	16.7%	1539 (0.2)	753,570 (99.8)		
Mixed Race	127,388	2.8%	311 (0.2)	126,918 (99.8)		
Other Non-White	274,327	6.0%	596 (0.2)	273,223 (99.8)		
White	3,051,298	67%	8729 (0.3)	3,035,422 (99.7)		
Missing	57,915	1.3%				
Body mass index of mother						
Underweight (<18.5)	141,959	3.1%	494 (0.3)	141,200 (99.7)	99.03	<0.001
Normal (18.5–24.9)	1,898,401	41.8%	5518 (0.3)	1,888,558 (99.7)		
Overweight (25.0–29.9)	1,130,903	24.9%	2711 (0.2)	1,124,409 (99.8)		
Obese (30+)	1,151,785	25.4%	3053 (0.3)	1,146,051 (99.7)		
Missing	213,819	4.7%				
Mothers who are smokers Regardless of pregnancy status.						
Yes	311,557	6.9%	1876 (0.6)	308,897 (99.4)	1375.41	<0.001
No	4,203,389	92.6%	10,301 (0.2)	4,183,100 (99.8)		
Missing	21,921	0.5%				
Smoking only during pregnancy						
Yes	327,826	7.2%	10,286 (0.2)	4,118,488 (99.8)	1032.85	<0.001
No	4,138,986	91.2%	1807 (0.6)	324,968 (99.4)		
Missing	70,055	1.5%				
Women who reported depression regardless of pregnancy status						
Yes	668,354	14.7%	1922 (0.3)	664,596 (99.7)	7.47	<0.006
No	3,823,333	84.3%	10,277 (0.3)	3,803,706 (99.7)		
Missing	45,179	1.0%				
Depression only during pregnancy						
Yes	64,2407	14.2%	2377 (0.4)	638,081 (99.6)	261.7	<0.001
No	3,808,470	83.9%	9765 (0.3)	3,789,375 (99.7)		
Missing	85,990	1.9%				
Mothers reported anxiety regardless of pregnancy status						
Yes	487,461	10.7%	1086 (0.2)	485,238 (99.8)	295.1	<0.001
No	3,409,226	75.1%	9974 (0.3)	3,390,314 (99.7)		
Missing	640,180	14.1%	1158 (0.2)	637,796 (99.8)		
Abuse only during pregnancy						
Yes	63,450	1.4%	264 (0.4)	63,029 (99.6)	51.2	<0.001
No	4,392,556	96.8%	11,757 (0.3)	4,369,716 (99.7)		
Missing	80,861	1.8.%				
Mothers who reported diabetes regardless of pregnancy status						
Yes	141,146	3.1%	217 (0.2)	140,473 (99.8)	73.65	<0.001
No	4,344,290	95.8%	11,925 (0.3)	4,321,576 (99.7)		
Missing	51,431	1.1%				
Diabetes only during pregnancy						
Yes	434,979	9.6%	1259 (0.3)	432,752 (99.7)	5.16	0.023
No	4,036,773	89.0%	10,918 (0.3)	4,015,612 (99.7)		
Missing	65,114	1.4%				
Vitamin intake						
Folic acid						
Yes	2,238,514	49.3%	5595 (0.3)	2,227,582 (99.7)	135.45	<0.001
No	1,751,954	38.6%	4764 (0.3)	1,743,099 (99.7)		
Missing	546,399	12.0%	1860 (0.3)	542,667 (99.7)		
Hypertension						
Yes	455,088	10%	1453 (0.3)	452,020 (99.7)	49.96	<0.001
No	4,074,956	89.8%	10,765 (0.3)	4,055,870 (99.7)		
Missing	6823	0.2%				

Note: Significant level *p* ≤ 0.05; ^†^ *p*-value based on the Chi-square test. ^‡^ N = Sample size ^§^ Chi-square = Chi-square test value.

**Table 2:** The risk evaluation of smoking, diabetes, and depression during pregnancy on birth defects, United States, 2018.

	*p*-Value ^†^	Odds Ratio	95% CI ^‡^
Maternal Age	<0.001	.	
<=17	0.367	0.86	(0.62–1.20)
18–19	<0.001	1.67	(1.49–1.87)
20–24	<0.001	1.24	(1.17–1.32)
25–29	1		
30–34	0.214	0.97	(0.91–1.02)
35–39	0.525	0.98	(0.92–1.05)
40+	<0.001	**2.20**	(2.01–2.40)
Maternal Race	<0.001		
White	1		
Asian	<0.001	**1.17**	(1.07–1.28)
Other American Including Tribes	<0.001	0.61	(0.47–0.80)
Black	<0.001	0.74	(0.69–0.79)
Mixed Race	0.895	0.99	(0.88–1.12)
Other Non-White	0.001	0.83	(0.75–0.93)
Income	<0.001		
Zero to USD28,000	0.094	0.95	(0.90–1.01)
USD 28,000 to 57,000	0.012	1.08	(1.02–1.14)
USD 57,000 to 85,000 or More	1		
Vitamin	<0.001		
Everyday/Week	1		
Didn’t Take Vitamin	<0.001	0.81	(0.77–0.85)
1–3 Times/Week	0.648	0.98	(0.91–1.06)
4–6 Times/Week	<0.001	0.83	(0.76–0.91)
Hypertension			
No	1		
Yes	<0.001	1.28	(1.20–1.36)
Body mass index of Mother	<0.001		
Normal (18.5–24.9)	1		
Underweight (<18.5)	<0.001	1.41	(1.28–1.55)
Overweight (25.0–29.9)	<0.001	0.82	(0.78–0.86)
Obese (30.0+)	0.005	1.08	(1.02–1.13)
Abuse only during pregnancy			
No	1		
Yes	0.175	1.11	(0.96–1.29)
Mothers who are smokers regardless of pregnancy status.			
No	1		
Yes	<0.001	2.29	(2.09–2.51)
Mothers who reported diabetes regardless of pregnancy status			
No	1		
Yes	<0.001	0.31	(0.25–0.39)
Mothers who reported depression regardless of pregnancy status			
No	1		
Yes	<0.001	0.58	(0.53–0.63)
Diabetes only during pregnancy			
No	1		
Yes	<0.001	0.75	(0.68–0.82)
Depression only during pregnancy			
No	1		
Yes	<0.001	1.31	(1.20–1.42)
Smoking only during pregnancy			
No	1		
Yes	<0.001	0.68	(0.60–0.77)
Folic acid intake	<0.001		
Yes	1		
Missing	<0.001	1.42	(1.33–1.51)
No	<0.001	1.16	(1.11–1.22)
Mothers reported anxiety regardless of pregnancy status	<0.001		
No	1		
Missing	<0.001	0.68	(0.63–0.72)
Yes	<0.001	0.75	(0.70–0.81)
Smoking ^1^ * depression ^2^ * diabetes ^3^			
No	1		
Yes	0.966	0	0
Smoking ^1^ * depression ^2^			
No	1		
Yes	<0.001	3.17	(2.76–3.64)
Smoking ^1^ * diabetes ^3^			
No	1		
Yes	<0.001	0.21	(0.10–0.44)
Depression ^2^ * diabetes ^3^			.
No	1		
Yes	<0.001	3.13	(2.67–3.66)
Constant	0	0.003	

Note: Significant level *p* ≤ 0.05 based on multivariate logistic regression model. * = represent interaction of two variable ^†^ *p*-value of odds ratio based on the multivariate logistic regression model. ^‡^ CI = Confidence Interval; 1 = Smoking only during pregnancy; 2 = depression only during pregnancy; 3 = diabetes only during pregnancy.

## Data Availability

Restrictions apply to the availability of these data. Data were obtained from CDC-PRAMS and are available (https://www.cdc.gov/prams/prams-data/researchers.htm (accessed on 6 January 2021)) with the permission of CDC-PRAMS.

## References

[1] Data & Statistics on Birth Defects|CDC Centers for Disease Control and Prevention. 23 January 2020. https://www.cdc.gov/ncbddd/birthdefects/data.html.

[2] Strauss R.P., Cassell C.H. (2009). Critical Issues in Craniofacial Care: Quality of Life, Costs of Care, and Implications of Prenatal Diagnosis. Acad. Pediatr..

[3] Moore L.L., Singer M.R., Bradlee M.L., Rothman K.J., Milunsky A. (2000). A Prospective Study of the Risk of Congenital Defects Associated with Maternal Obesity and Diabetes Mellitus. Epidemiology.

[4] Behavioral Risk Factor Surveillance System 2019 Summary Data Quality Report July 16, 2020. July 2020. https://www.cdc.gov/brfss/annual_data/2019/pdf/2019-sdqr-508.pdf.

[5] Hackshaw A., Rodeck C., Boniface S. (2011). Maternal smoking in pregnancy and birth defects: A systematic review based on 173 687 malformed cases and 11.7 million controls. Hum. Reprod. Updat..

[6] Impellizzeri A., Giannantoni I., Polimeni A., Barbato E., Galluccio G. (2019). Epidemiological characteristic of Orofacial clefts and its associated congenital anomalies: Retrospective study. BMC Oral Health.

[7] Ban L., Gibson J., West J., Fiaschi L., Sokal R., Smeeth L., Doyle P., Hubbard R., Tata L. (2014). Maternal Depression, antidepressant prescriptions, and congenital anomaly risk in offspring: A population-based cohort study. BJOG Int. J. Obstet. Gynaecol..

[8] Yu H., Jiang X., Bao W., Xu G., Yang R., Shen M. (2018). Association of intimate partner violence during pregnancy, prenatal depression, and adverse birth outcomes in Wuhan, China. BMC Pregnancy Childbirth.

[9] Shulman H.B., D’Angelo D.V., Harrison L., Smith R.A., Warner L. (2018). The Pregnancy Risk Assessment Monitoring System (PRAMS): Overview of Design and Methodology. Am. J. Public Health.

[10] Chisnall P.M. (2007). Mail and Internet Surveys: The Tailored Design Method. J. Advert. Res..

[11] PRAMS Methodology|CDC (2019). CDC-PRAMS. https://www.cdc.gov/prams/methodology.htm#2.

[12] Steinbacher D., Sierakowski S. (2012). First Aid for the NBDE Part 1.

[13] Berg E., Lie R.T., Sivertsen S., Haaland Y.A. (2015). Parental age and the risk of isolated cleft lip: A registry-based study. Ann. Epidemiol..

[14] Rodríguez A. (2010). Risk factors associated with metabolic syndrome in type 2 diabetes mellitus patients according to World Health Organization, Third Report National Cholesterol Education Program, and International Diabetes Federation definitions. Diabetes Metab. Syndr. Obes. Targets Ther..

[15] Biggio J.R., Chapman V., Neely C., Cliver S.P., Rouse D.J. (2010). Fetal Anomalies in Obese Women. Obstet. Gynecol..

[16] Xuan Z., Zhongpeng Y., Yanjun G., Jiaqi D., Yuchi Z., Bing S., Chenghao L. (2016). Maternal active smoking and risk of oral clefts: A meta-analysis. Oral Surg. Oral Med. Oral Pathol. Oral Radiol..

[17] Shelke G.S., Marwaha R.S., Shah P., Challa S. (2023). Role of Patient’s Ethnicity in Seeking Preventive Dental Services at the Community Health Centers of South-Central Texas: A Cross-Sectional Study. Dent. J..

[18] Home Visiting | MCHB. HRSA. 13 October 2021. https://mchb.hrsa.gov/programs-impact/programs/home-visiting.

[19] Pereira A.V., Fradinho N., Carmo S., de Sousa J.M., Rasteiro D., Duarte R., Leal M.J. (2018). Associated Malformations in Children with Orofacial Clefts in Portugal. Plast. Reconstr. Surg.-Glob. Open.

